# Cancer Prevention Using Machine Learning, Nudge Theory and Social Impact Bond

**DOI:** 10.3390/ijerph17030790

**Published:** 2020-01-28

**Authors:** Daitaro Misawa, Jun Fukuyoshi, Shintaro Sengoku

**Affiliations:** 1 Department of Innovation Science, School of Environment and Society, Tokyo Institute of Technology, Tokyo 152-8850, Japan; misawa.d.aa@m.titech.ac.jp; 2 Cancer Scan, Co., Ltd., Tokyo 141-0031, Japan; fukuyoshi@cancerscan.jp; 3 Life Style by Design Research Unit, Institute for Future Initiatives, the University of Tokyo, Tokyo 113-0033, Japan

**Keywords:** disease prevention, machine learning, nudge theory, medical data, social impact bond

## Abstract

There have been prior attempts to utilize machine learning to address issues in the medical field, particularly in diagnoses using medical images and developing therapeutic regimens. However, few cases have demonstrated the usefulness of machine learning for enhancing health consciousness of patients or the public in general, which is necessary to cause behavioral changes. This paper describes a novel case wherein the uptake rate for colorectal cancer examinations has significantly increased due to the application of machine learning and nudge theory. The paper also discusses the effectiveness of social impact bonds (SIBs) as a scheme for realizing these applications. During a healthcare SIB project conducted in the city of Hachioji, Tokyo, machine learning, based on historical data obtained from designated periodical health examinations, digitalized medical insurance receipts, and medical examination records for colorectal cancer, was used to deduce segments for whom the examination was recommended. The result revealed that out of the 12,162 people for whom the examination was recommended, 3264 (26.8%) received it, which exceeded the upper expectation limit of the initial plan (19.0%). We conclude that this was a successful case that stimulated discussion on potential further applications of this approach to wider regions and more diseases.

## 1. Introduction

### 1.1. Aim and Objectives

Based on the ranking of the occurrence of different types of cancer in Japan, the incidence rate of colorectal cancer (CRC) is fourth among cancers for men (115.9 ppm) and second for women (80.5 ppm) [1]. The mortality rate of CRC is the third highest of all cancers for men (42.9 ppm) and the highest for women (34.6 ppm) [1]. The CRC screening program, which uses the fecal occult blood test (FOBT), is a recognized effective approach, and is, thus, reinforced by the public sector to reduce incidence and mortality rates [2]. Despite the expected effectiveness of the preventive approach, CRC screening is yet to be fully implemented, and fails to be recognized as important by the recipients, with uptake levels remaining at low levels. Whilst more than 38% of the population participated in a leading study in Japan in 2013, only 18.1% of the citizens of the city of Hachioji participated in 2016 [3]. In this regard, this study adopted a preventive effort toward CRC based on the combination of machine learning using real-world data (RWD), nudge theory, and social impact bonds (SIB).

### 1.2. Machine Learning in Healthcare

At present, the applications of information and communication technology (ICT) in the medical and healthcare fields have progressed to a point where artificial intelligence (AI), including machine learning, is positioned at its core [4,5]. Machine learning used in the healthcare field can be categorized into three phases in accordance with the patient’s journey: Prevention, diagnosis, and treatment. Significantly, the Food and Drug Agency (FDA) of the United States of America recognized the effectiveness of medical devices equipped with machine learning (software as a medical Device, SaMD), and established three clinical factors as quality measures: Valid clinical association, analytical validation, and clinical validation. Amongst the phases, the diagnosis field probably utilizes the technology the most [6,7,8,9,10,11].

Machine learning models are being intensively developed for the diagnosis phase to deduce and detect cancer from various available sources of medical images. In particular, the following examples were highly successful: A better prediction of breast cancer with a deep learning model combined with a logistic regression model (area under the curve (AUC) = 0.70 vs. 0.62, respectively) in which the image data and assessment records of over 39,571 subjects were combined [12]; the detection of early stage stomach cancer using a convolutional neural network (CNN, detection rate = 82.8%) [13]; the deduction of the risk level of lung cancer using a CNN model (AUC = 0.94) [14]. Regarding the treatment phase, machine learning is most used in cancer therapeutics. For example, International Business Machines Corporation (IBM)’s Watson for Oncology combines the cancer treatment knowledge of first-line oncologists with the computational excellence of IBM Watson to help practitioners suggest appropriate and customized treatment options [15] (IBM: The International Business Machines Corporation is an American multinational information technology company). However, as described previously, applications in the disease prevention phase have been limited to a few cases in other therapeutic areas, such as acute renal impairment [16], whereas this approach would have the potential from a medical economics perspective to help patients avoid a significant cost burden and decrease in quality of life.

In cases of CRC, machine learning methods are reportedly used to substantially improve the accuracy of predicting cancer susceptibility, recurrence and survival rate [17,18], and can be explored in multidisciplinary approaches with cytohistological analysis [19] or whole-genome sequencing analysis [20]. However, limited attention has been paid to the diagnostic phase, i.e., detecting target populations for an effective recommendation of diagnosis in combination with existing RWD assets such as electric health records and medical receipts.

### 1.3. Nudge Theory towards Behavioral Changes

Nudge is a measure used to induce behavioral change by means of behavioral economics based on the notion of libertarian paternalism that secures subjects’ freedom of choice without providing any financial incentives [21]. In the field of health care, the trans-theoretical model forms a basis for applying nudge to health-influencing behaviors [22]. The model claims that health-influencing behavioral changes progress through the following 6 stages: Pre-contemplation, contemplation, preparation, action, maintenance, and termination. Notably, applied research demonstrates dramatic improvements in recruitment, retention, and progress using stage-matched interventions and proactive recruitment procedures. The study proposes that the most promising outcomes were found with computer-based, individualized, and interactive interventions, which predicted the effectiveness of personalized interventions in cancer prevention in the form of a nudge.

Despite several successful cases of ICT-driven behavioral changes in e-commerce, there are few cases known in the medical field, as medical practices, including diagnosis and treatment, must be approved by medical practitioners. Thus, it is difficult to promote behavioral changes in patients in a direct manner. In contrast, direct intervention may be possible during the prevention phase, which does not necessarily require professional intervention. There are several precedent cases, though few of them enhance behavioral change. One example is the SENIOR Project, which focused on 200 elderly patients with slight cognitive impairment. The application sent notifications designed using nudge theory based on patient profiles customized with machine learning [23]. This approach was expected to prevent the progression of mild cognitive impairment and improve overall health.

Nudge theory provides a methodological cue which produces a significant impact on behaviors by taking small actions, such as minor revision or customization of voice/text messages to a subject [24,25]. One example of this is the Human Behavior-Change Project (HBCP) organized by IBM [26]. The goal of the project was to develop an AI system, capable of extracting insights and developing predictions related to behavioral changes, for health promotion or disease prevention. Hornik presented another example [27]. This project recommended a strategy to develop text messages that effectively promote behavioral changes in the case of a smoking cessation-campaign, focusing on three viewpoints: (i) “Are there many people with the wrong message-relevant belief?”; (ii) “is there a substantial association between belief and outcome?”; and (iii) “can belief be affected by an educational campaign?” This approach was also applied to a precedent study conducted by the city of Hachioji in 2016 [2]. It revealed that a message for loss-avoidance based on prospect theory was more effective than a normal one.

### 1.4. Advanced Preventive Medicine Using Health Data

Health data can be broadly organized into four categories: Image, examination data including medical insurance receipts, genetic information, and text, such as articles/reports, dialogues, and medical interviews. As an example, image data is currently utilized to perform imaging-based diagnosis [12],genetic data is used to prescribe therapeutic regimens for cancer treatment, and text data is used for chat-based automated diagnosis [10]. The examination data that the present study focuses on is being proprietarily used to predict the worsening of conditions and the onset of diseases [28]. As a symbolic case in this category, health examination data used to predict the risk of stomach cancer included a customized dataset that consisted of biological characteristics, infection conditions of Helicobacter pylori, endoscopic diagnosis, and blood testing in order to conduct XGboost learning. This resulted in the successful prediction of disease onset with an accuracy of AUC 0.899 [29]. In another case study, the use of machine learning based on individual treatment histories as presented by digitalized medical insurance receipt data, predicted the onset of Alzheimer’s disease with an AUC of 0.730. This study had a comparatively higher accuracy than the existing models, which used the results of the analyses of cerebrospinal fluid (CSF), blood samples, cognitive tests, and brain image diagnosis [30]. In particular, the government of Japan launched its designated cancer examination system in 2008 and accumulated a large amount of related health record data which was utilized in the present study.

### 1.5. Social Impact Bond in Healthcare

**Figure 1:**
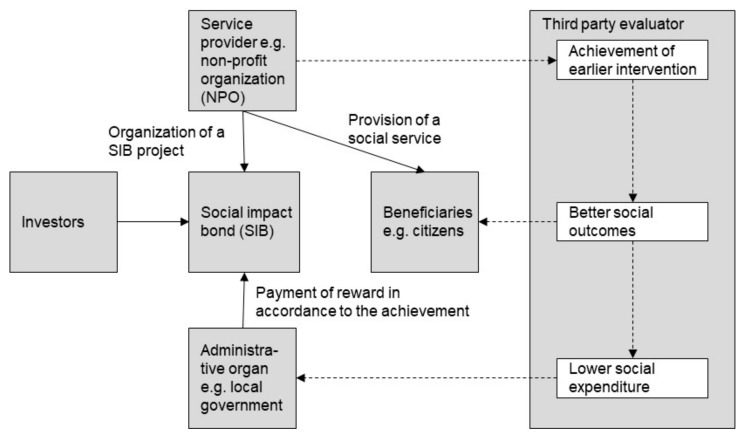
General structure and scheme of a social impact bond (SIB).

The SIB is a contract with the public sector or governing authority which pays for better social outcomes in certain areas and allocates part of the savings achieved to investors [31]. A SIB is a type of bond; however, it has a unique characteristic—the organizer of a SIB project operates the project over a fixed period, without determining a fixed rate of return in advance. Instead, repayment to investors is contingent upon the achievement of specified social outcomes. Therefore, in terms of investment risk, SIBs have a similar characteristic to a structured product or an equity investment. Figure 1 illustrates the general structure and scheme of a SIB.

The world’s first SIB began in 2010, Peterborough, England, to prevent repeat criminal offenses and facilitate former offenders’ return to society [32]. However, SIB projects are not limited to preventing repeat offenses; there are various other types of projects, such as youth employment support, homeless independence support, childcare, early childhood education, and healthcare. Although 137 SIBs are currently being implemented in 25 countries, workforce development and housing for homeless people are the focus of more than half of these projects [33].

11 SIB projects were conducted in the healthcare field as of March 2019 [34]. Surprisingly, none of the projects used a standard cost-quality measure in medical and healthcare practices. The quality-adjusted life year (QALY) is a generic measure of disease burden, including both the quality and quantity of life, and is thus widely used in economic evaluation to assess the monetary value of medical interventions. This measure had not been used in the above-mentioned SIB projects, although it can be effective in evaluating the projects. Moreover, the objectives of all these SIB projects, except one case which will be described in the next section, were merely oriented towards cost-saving, i.e., there was no objective to improve the quality of life of the patients.

## 2. Materials and Methods

### 2.1. The Case

This paper presents the novel case of significantly increasing the diagnostic rate of CRC by applying a combination of machine learning, nudge theory and SIB. As technical approach, machine learning identifies the target segment, whilst nudge theory leads the behavioral changes of the recipients towards actual diagnosis. The SIB works well as a scheme for implementing these approaches for a municipality which is risk-averse to adopting novel approaches involving initial investment. The case project was introduced by the city of Hachioji, Tokyo, Japan in 2017 in collaboration with Cancer Scan Co., Ltd.; K-three Inc.; Japan Social Impact Investment Foundation (SIIF); DIGISEARCH & ADVERTISING, Inc.; and Mizuho Bank, Ltd. [3]. The project achieved the improvement of outcome-based CRC screening examinations. Hachioji city was the hosting organization; Cancer Scan and K-three were the technology and service providers, respectively; and SIIF, DIGISEARCH & ADVERTISING and Mizuho Bank were financial investors in the SIB scheme.

### 2.2. Dataset

Records of designated health examinations for citizens over the age of 40, track records of CRC examinations sponsored by the city of Hachioji, and digitalized medical insurance receipt data over a six year-period (2010–2016) were used as the initial dataset of the current study. The designated health examination data included the attributes of the recipients, such as age, gender, height, and weight; biochemical testing data, such as levels of aspartate transaminase (AST), HbA1c, and total cholesterol (TC) in blood; and results of medical interviews on smoking and medication. The CRC examination data included information on the examination history and subsequent results. Digital medical insurance receipt data of people in the city of Hachioji included the subjects’ histories of examination diagnosis and prescription.

### 2.3. Machine learning model

Based on the aforementioned data, Cancer Scan conducted supervised learning using Light Gradient Boosting Machine (LGBM, Microsoft Corporation) [35]. In comparison to other machine learning methods, in particular deep learning, LGBM is considered to be suitable for data with clear feature values such as test values and lifestyle. The outcome variable was defined to be the rate of uptake of CRC screening in 2016 and subjected to training and test data where track records of CRC screening over the past five years (2011–2015), gender, and age were assigned to the covariates. The hyper-parameters for the machine learning approach were determined through Bayesian optimization. Confidence scores were used instead of cut-off values to select target subjects for recommendation according to the order of the scores. Python 3 (The Python Software Foundation, Delaware, USA), a programming language, was applied to these procedures.

### 2.4. Recommendation Materials

**Figure 2:**
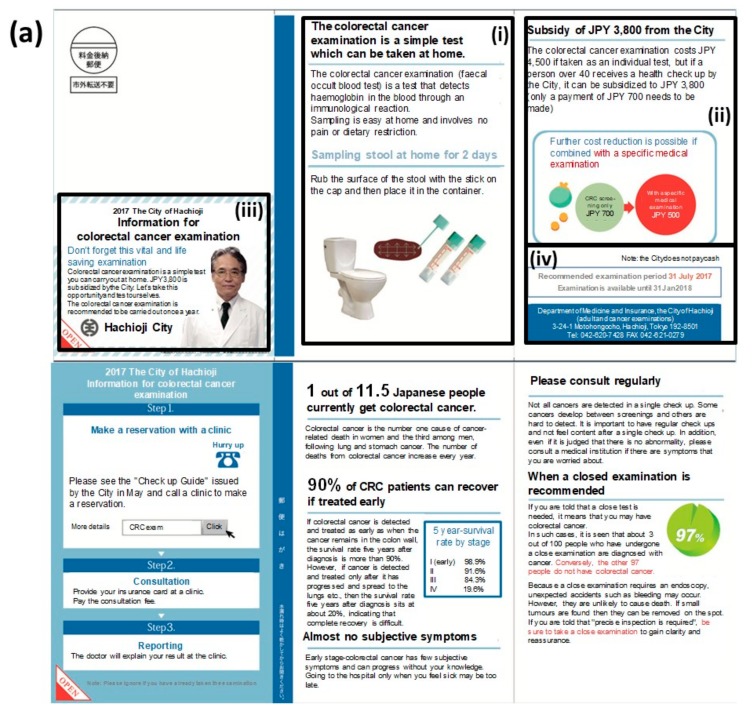

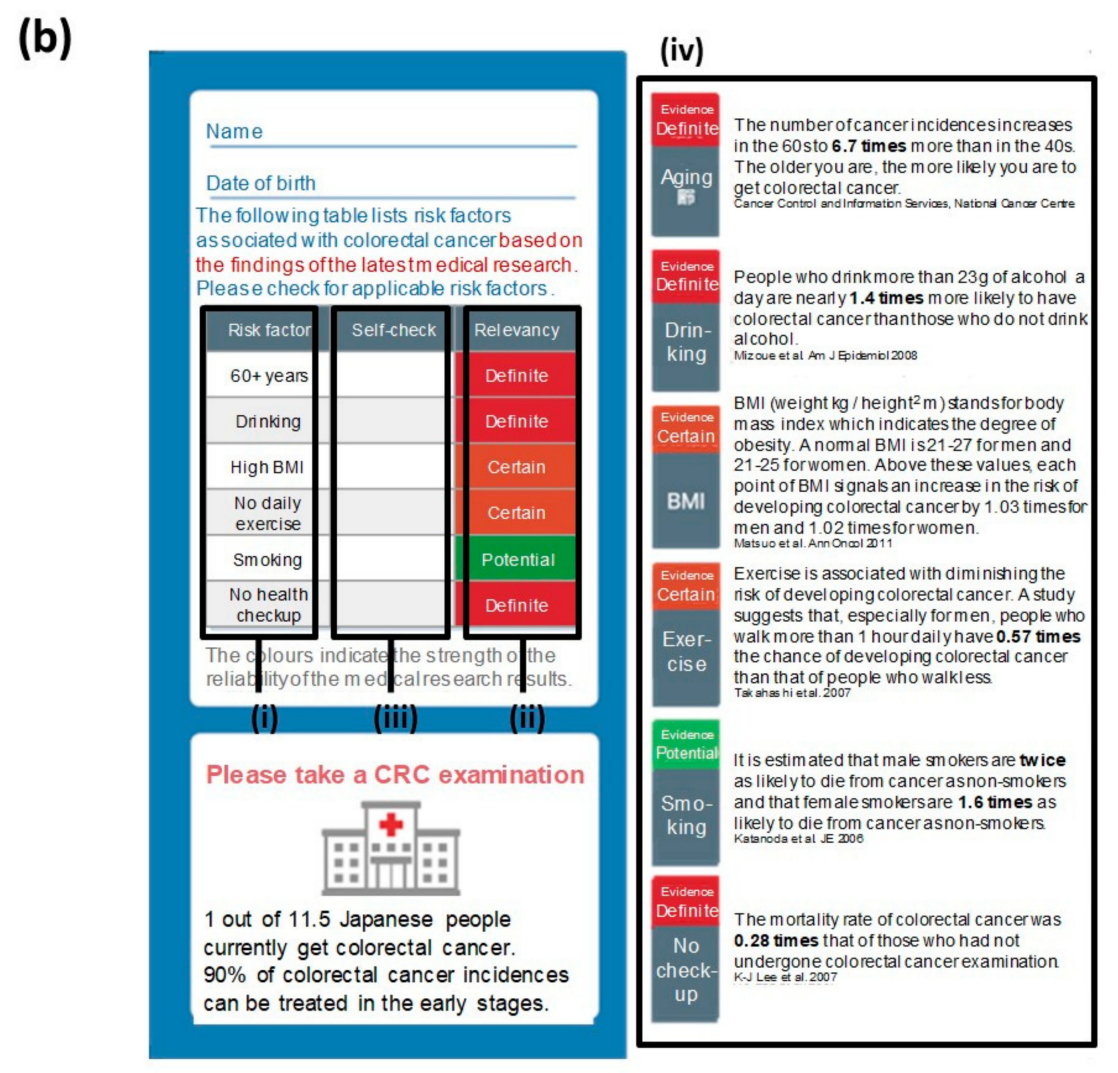

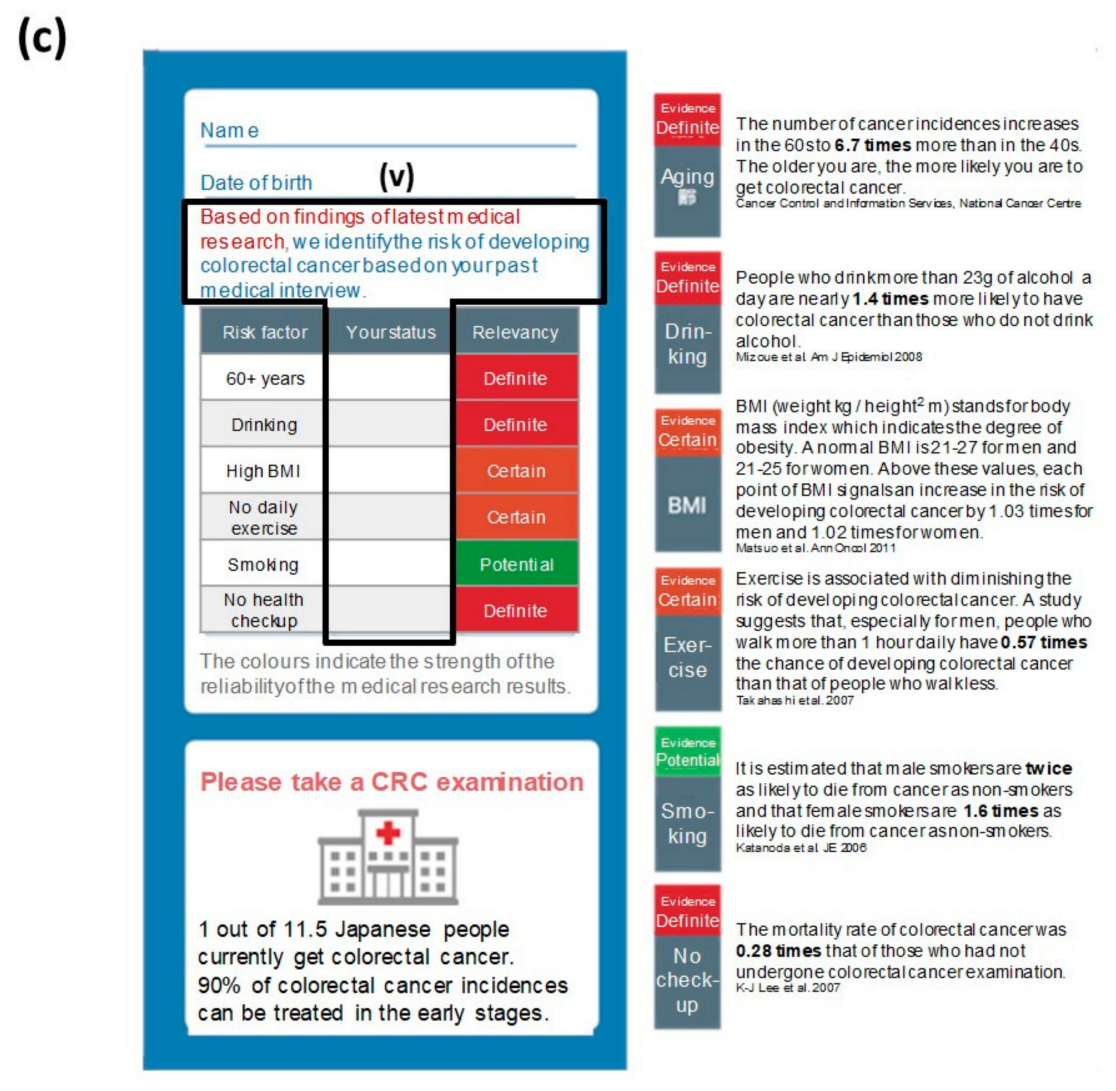
The recommendation materials used in this case. (**a**) The common material of the first round of recommendations contained each piece of the ‘EAST (easy, attractive, society, and timely)’ framework for nudge: (i) It emphasized that CRC screening can be self-completed at home (easy); (ii) it mentioned that the subsidy from the city would reduce the actual cost burden by JPY 3800 (84.5%; attractive); (iii) it was clearly indicated that it was a notification from the city for a public health purpose (society); (iv) it set a fixed period for CRC screening with a defined deadline (timely). (**b**) The non-customized material in the second round of recommendations exhibited a check sheet with (i) the major six risk factors of CRC, (ii) the relevancy of each factor to the incidence colored in red, orange and green in accordance to the risk level, (iii) the relevancy of the recipient’s condition to each of these factors for self-checking, and (iv) epidemiological details in each of these factors. (**c**) The customized material in the second round of recommendations used an identical format to the one in (**c**) except (v) the relevancy of the recipient’s condition that had already been filled in by the sender based on a precedent medical consultation. Note: These images or descriptions were translated into English by the authors. Supplementary figure provides the original version in Japanese. Note: These figures were translated by the authors from the original materials (Supplementary Figure S1).

A call/recall process was adopted by sending the recommendation material twice. In preparing recommendation materials, a framework called ‘EAST’ was adopted to create nudge-based materials (Figure 2) [36,37]. The EAST raises four points that are effective in behavioral changes: Easy, attractive, social, and timely. The recommendation material used in the first round (Figure 2a) was designed to equally cover these four points, whereas the material for the second round (Figure 2b,c) were oriented towards a subject recognizing the importance of taking CRC screening as a personal matter by showing the potential risk factor of the recipient in accordance to the following six risk factors relevant to CRC: (i) Over the age of 60, (ii) drinking alcohol regularly, (iii) high body mass index, (iv) lack or marginal level of exercise, (v) smoking, and (vi) not receiving periodic medical examinations.

### 2.5. Ethical Considerations

The present study did not require an institutional review board (IRB) approval according to the following reasons: (i) The City of Hachioji conducted this initiative for CRC prevention as part of its ordinary service for health promotion and welfare, fully complying with the national and municipal regulations; (ii) Cancer Scan Co., Ltd. was an outsourced operator as part of this initiative and dedicated only to developing an algorithm to select target segments for health recommendation based on an anonymized and decoupled dataset provided by the City. The IRB process was not required in the contact produced by the City nor by the country regulation; (iii) the Tokyo Institute of Technology has not received any dataset related to this initiative.

## 3. Results

Of the 102,501 National Health Insurance subscribers in the city of Hachioji, 74,069 citizens who had not undergone CRC screening in the previous year were screened out. Then, the machine learning model was trained with the above-mentioned dataset to obtain an AUC of 0.8793. Each of the confidence scores was inferred using the trained machine learning model for all target individuals to visit the CRC screening. As a result, 12,162 individuals were selected using the selection criteria as described above for the first round of recommendations in June 2017 using the common material (Figure 2a). As a result, 1255 recipients (10.3%) took CRC screening.

**Figure 3:**
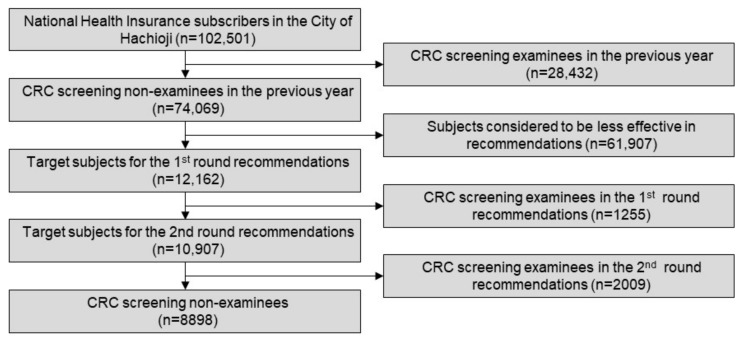
A consort diagram of the procedure.

The second round of recommendations was sent to the residual 10,907 subjects six months after the first round. In this round, two types of recommendations were applied. Recommendations using the common (non-customized) material (Figure 2b) were used for 5548 subjects, whereas the customized ones (Figure 2c) were used for the other 5359. These subjects were assigned to either of these two groups in accordance to an aggregate result that the city of Hachioji had organized from each hospital or clinic. As a result, an additional 2009 subjects (18.4%) started taking CRC screening. Through these procedures, the interim result acquired 3264 recipients in total for CRC screening (26.8%; Figure 3), which far exceeded the goal of 19.0% initially set by the organizer, resulting in the full pay-for-performance amount to the operator throughout the SIB scheme.

## 4. Discussion

Increasing the rate of cancer screening, especially CRC, is one of the most important challenges for preventive medicine in Japan for the following reasons: (i) The high mortality rates of common cancer strains (first for women and third for men in Japan), (ii) strong endorsement by the national guidelines, (iii) the simplicity of the initial screening using FOBT and the promptness of further examination by an endoscopic approach, finally resulting in (iv) an optimal cost-benefit profile in the designed intervention.

The superiority of the present approach as compared with existing initiatives can be summarized as follows. First, the given case was the first trial in Japan that applied an RWD-based machine learning approach to a city-wide real example of cancer prevention. As mentioned previously, there are various applications of machine learning currently in use in the diagnosis and treatment phases; however, they are rare in the prevention phase. The present study succeeded in utilizing this method to identify a target segment of citizens who can most effectively be nudged towards CRC screening. Furthermore, in this study we conducted an integrated operation using both medical and health information, whereas medical examinations conducted by municipalities were often disconnected from medical practices. In this case, a variety of information sources were utilized, including demographic information of the citizens such as age, gender and living environment, medical information such as diagnostic and treatment histories, and health information such as health check-up data. As all municipalities in Japan use designated health examinations and cancer screenings using a defined and identical format, this could be an ideal environment to deploy the approach to wider geographical conditions.

Second, the SIB scheme was considered to be essential for the smooth implementation of these novel approaches to overcome the municipality’s conservative attitude towards novel approaches and a reluctance for long-term investment. The structuring process significantly contributed to the visualization of which business implementation effects could be expected and to what extent medical expenses could be optimized. As a result, the municipality as a national health insurer can directly optimize the medical expenses for the target citizens. In addition, with the SIB scheme a more efficient risk-benefit profile was developed for the municipality. In particular, a limited initial financial investment was required to initiate the program, and no payment would have been required if the efforts would not have reached the outcome goal resulting in appropriate allocation of medical resources. Furthermore, the SIB scheme is open to a wide range of operators who have effective skills and knowledge, and investors who agree on its social value.

Finally, several limitations should be described. The successful result of the present approach was favored by the above-mentioned conditions which are specific to CRC. In other words, the applicability to other types of cancer should be carefully examined and the method optimized accordingly. This trial presented three types of interventions as described above. Because of the case setting in a municipal administration and restrictions on the Personal Information Protection Law in Japan, the subject-related information used in the study can be partially disclosed. It is difficult to conclusively state whether this research design was suitable for verifying results for which the goal was to increase the rate at which subjects received examinations. Statistical analysis using propensity scores would likely be required to accurately verify the obtained results. It would be beneficial to conduct an additional trial through a randomized control study to verify the results of the subject selection. Based on these considerations and further needs for improvement, the proposed approach is expected to be expanded to become an effective and feasible way to help the public sector lead social innovation in the prevention of CRC and other diseases.

## 5. Conclusions

The present paper discusses a method for improving the uptake-rate of citizens receiving CRC examinations where a combination of machine learning, nudge theory and a SIB scheme was applied. Notably, 3264 (26.8%) of the 12,162 subjects who were recommended CRC screening actually received the examination, which significantly exceeded the initial expectation (19.0%). Our observation reveals the effectiveness of RWD-based machine learning for improving the detection of target segments of the population, an application of nudge theory for more effective recommendations for taking health examinations, an SIB scheme for the smoother implementation of these novel approaches, and the effectiveness of a combined operation of these three components in the real case of preventive medicine. We conclude that this study served as a successful case to open discussions on further applications of this combined approach to wider regions and diseases.

## Supplementary Materials

The following are available online at https://www.mdpi.com/1660-4601/17/3/790/s1, Figure S1: The original recommendation materials of Figure 2.
