# Supplementary material for: Cancer Prevention Using Machine Learning, Nudge Theory and Social Impact Bond
Source: Int J Environ Res Public Health. 2020 Jan 28;17(3):790. doi: 10.3390/ijerph17030790 (PMC7037430; doi:10.3390/ijerph17030790)
Supplement: Supplementary file 1 [file ijerph-17-00790-s001.pdf]

Supplementary Material

(a)

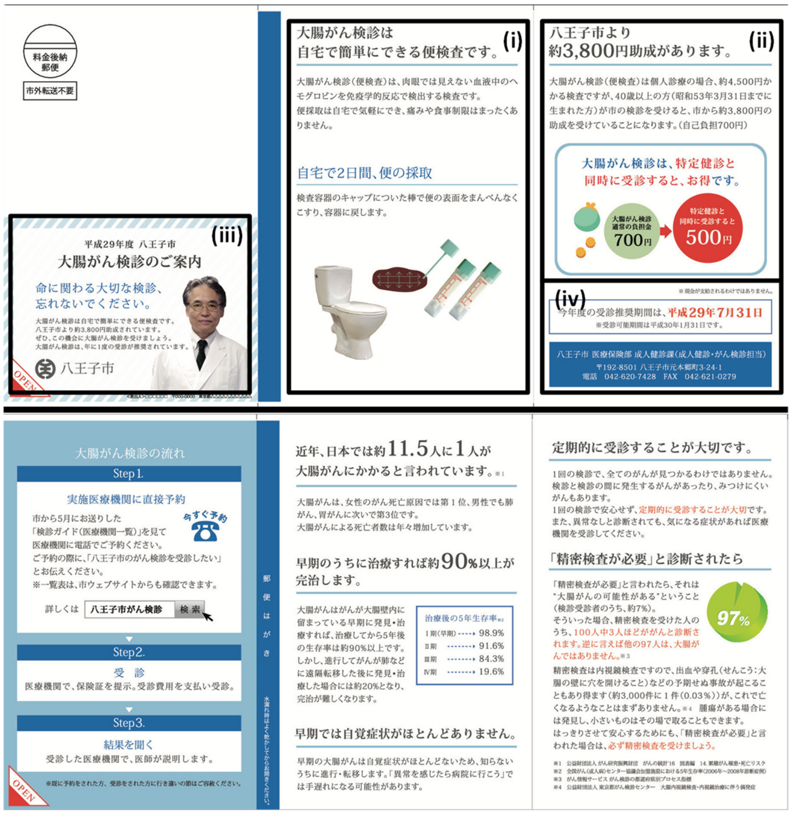

(b)

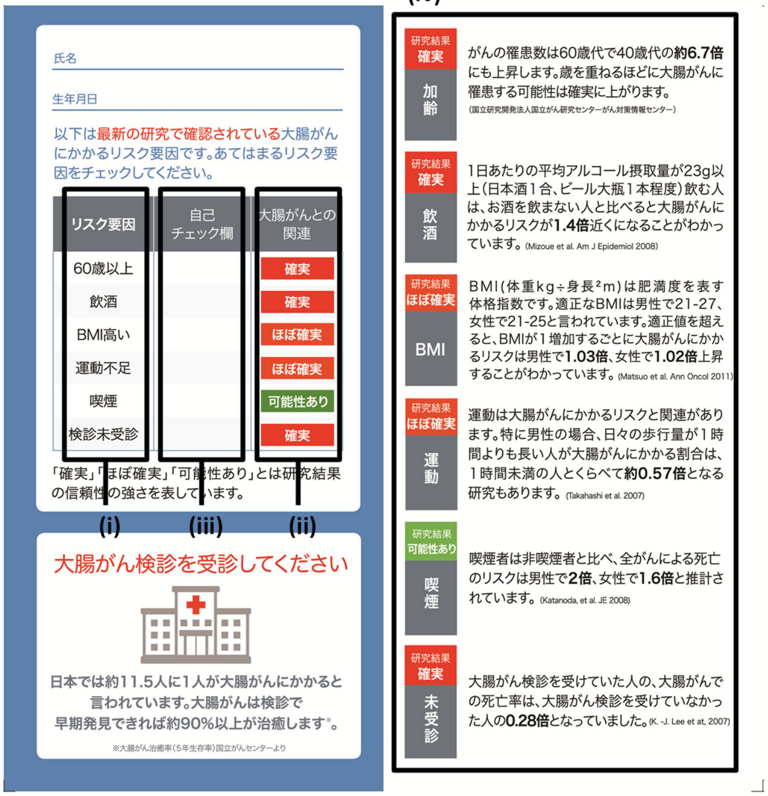
(iv)
氏名
生年月日
以下は最新の研究で確認されている大腸がんにかかるリスク要因です。あてはまるリスク要因をチェックしてください。
リスク要因
自己チェック欄
大腸がんとの関連
60歳以上
飲酒
BMI高い
運動不足
喫煙
検診未受診
確実
確実
ほぼ確実
ほぼ確実
可能性あり
確実
「確実」「ほぼ確実」「可能性あり」とは研究結果の信頼性の強さを表しています。
(i)
(iii)
(ii)
大腸がん検診を受診してください
日本では約11.5人に1人が大腸がんにかかると言われています。大腸がんは検診で早期発見できれば約90%以上が治癒します※。
※大腸がん治癒率(5年生存率)国立がんセンターより
研究結果 確実 加齢
がんの罹患数は60歳代で40歳代の約6.7倍にも上昇します。歳を重ねるほどに大腸がんに罹患する可能性は確実に上がります。
(国立研究開発法人国立がん研究センターがん対策情報センター)
研究結果 確実 飲酒
1日あたりの平均アルコール摂取量が23g以上(日本酒1合、ビール大瓶1本程度)飲む人は、お酒を飲まない人と比べると大腸がんにかかるリスクが1.4倍近くになることがわかっています。(Mizoue et al. Am J Epidemiol 2008)
研究結果 ほぼ確実 BMI
BMI(体重kg÷身長²m)は肥満度を表す体格指数です。適正なBMIは男性で21-27、女性で21-25と言われています。適正値を超えると、BMIが1増加するごとに大腸がんにかかるリスクは男性で1.03倍、女性で1.02倍上昇することがわかっています。(Matsuo et al. Ann Oncol 2011)
研究結果 ほぼ確実 運動
運動は大腸がんにかかるリスクと関連があります。特に男性の場合、日々の歩行量が1時間よりも長い人が大腸がんにかかる割合は、1時間未満の人とくらべて約0.57倍となる研究もあります。(Takahashi et al. 2007)
研究結果 可能性あり 喫煙
喫煙者は非喫煙者と比べ、全がんによる死亡のリスクは男性で2倍、女性で1.6倍と推計されています。(Katanoda, et al. JE 2008)
研究結果 確実 未受診
大腸がん検診を受けていた人の、大腸がんでの死亡率は、大腸がん検診を受けていなかった人の0.28倍となっていました。(K.-J. Lee et at, 2007)

(c)

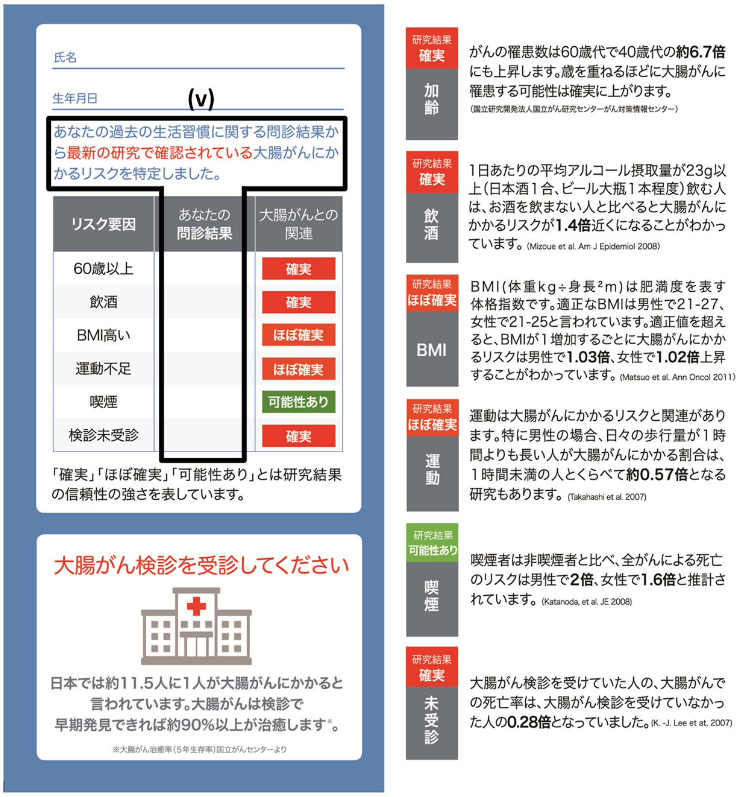
氏名
生年月日
(v)
あなたの過去の生活習慣に関する問診結果から最新の研究で確認されている大腸がんにかかるリスクを特定しました。
リスク要因
あなたの問診結果
大腸がんとの関連
60歳以上
飲酒
BMI高い
運動不足
喫煙
検診未受診
確実
確実
ほぼ確実
ほぼ確実
可能性あり
確実
「確実」「ほぼ確実」「可能性あり」とは研究結果の信頼性の強さを表しています。
大腸がん検診を受診してください
日本では約11.5人に1人が大腸がんにかかると言われています。大腸がんは検診で早期発見できれば約90%以上が治癒します※。
※大腸がん治癒率(5年生存率)国立がんセンターより
研究結果 確実 加齢
がんの罹患数は60歳代で40歳代の約6.7倍にも上昇します。歳を重ねるほどに大腸がんに罹患する可能性は確実に上がります。
(国立研究開発法人国立がん研究センターがん対策情報センター)
研究結果 確実 飲酒
1日あたりの平均アルコール摂取量が23g以上(日本酒1合、ビール大瓶1本程度)飲む人は、お酒を飲まない人と比べると大腸がんにかかるリスクが1.4倍近くになることがわかっています。(Mizoue et al. Am J Epidemiol 2008)
研究結果 ほぼ確実 BMI
BMI(体重kg÷身長²m)は肥満度を表す体格指数です。適正なBMIは男性で21-27、女性で21-25と言われています。適正値を超えると、BMIが1増加するごとに大腸がんにかかるリスクは男性で1.03倍、女性で1.02倍上昇することがわかっています。(Matsuo et al. Ann Oncol 2011)
研究結果 ほぼ確実 運動
運動は大腸がんにかかるリスクと関連があります。特に男性の場合、日々の歩行量が1時間よりも長い人が大腸がんにかかる割合は、1時間未満の人とくらべて約0.57倍となる研究もあります。(Takahashi et al. 2007)
研究結果 可能性あり 喫煙
喫煙者は非喫煙者と比べ、全がんによる死亡のリスクは男性で2倍、女性で1.6倍と推計されています。(Katanoda, et al. JE 2008)
研究結果 確実 未受診
大腸がん検診を受けていた人の、大腸がんでの死亡率は、大腸がん検診を受けていなかった人の0.28倍となっていました。(K.-J. Lee et at, 2007)

Figure S1. The original recommendation materials of Figure 2.
